# *Euphorbia* Factor L2 ameliorates the Progression of K/BxN Serum-Induced Arthritis by Blocking TLR7 Mediated IRAK4/IKKβ/IRF5 and NF-kB Signaling Pathways

**DOI:** 10.3389/fphar.2021.773592

**Published:** 2021-12-03

**Authors:** Jing Tang, Xiaolan Cheng, Shiyu Yi, Yuanyuan Zhang, Zhigang Tang, Yutong Zhong, Qiuping Zhang, Bin Pan, Yubin Luo

**Affiliations:** ^1^ Laboratory of Rheumatology and Immunology, West China Hospital, Sichuan University, Sichuan, China; ^2^ Department of Rheumatology and Immunology, Luzhou's People’s Hospital, Luzhou, China; ^3^ Affiliated Hospital of Integrated Traditional Chinese and Western Medicine, Nanjing University of Chinese Medicine, Nanjing, China; ^4^ Department of Rheumatology and Immunology, The General Hospital of Western Theater Command PLA, Chengdu, China; ^5^ Sichuan Food and Drug Inspection and Testing Institute, Chengdu, China; ^6^ Shandong Peninsula Engineering Research Center of Comprehensive Brine Utilization, Weifang University of Science and Technology, Shouguang, China

**Keywords:** euphorbia factor L2, serum-induced arthritis, TLR7, IRF5, rheumatoid arthritis

## Abstract

Toll like receptor (TLR)s have a central role in regulating innate immunity and their activation have been highlighted in the pathogenesis of rheumatoid arthritis (RA). EFL2, one of diterpenoids derived from *Euphorbia* seeds, is nearly unknown expect for its improving effect on acute lung injury. Our present study aimed to investigate EFL2’s pharmacokinetic features, its therapeutic effect on rheumatoid arthritis, and explored the potential anti-arthritic mechanisms. K/BxN serum transfer arthritis (STA) murine model was used to assess EFL2’s anti-arthritic effects. We also applied UPLC-MS method to measure the concentrations of EFL2 in plasma. The inhibitory effects of this compound on inflammatory cells infiltration and activation were determined by flow cytometry analysis and quantitative real-time polymerase chain reaction (qRT-PCR) *in vivo*, and immunochemistry staining and ELISA in murine macrophages and human PBMCs *in vitro*, respectively. The mechanism of EFL2 on TLRs mediated signaling pathway was evaluated by PCR array, Western blot, plasmid transfection and confocal observation. Intraperitoneal (i.p.) injection of EFL2, instead of oral administration, could effectively ameliorate arthritis severity of STA mice. The inflammatory cells migration and infiltration into ankles were also significantly blocked by EFL2, accompanied with dramatically reduction of chemokines mRNA expression and pro-inflammatory cytokines production. *In vivo* PCR microarray indicated that EFL2 exerted anti-arthritis bioactivity by suppressing TLR7 mediated signaling pathway. *In vitro* study confirmed the inhibitory effects of EFL2 on TLR7 or TLR3/7 synergistically induced inflammatory cytokines secretion in murine macrophages and human PBMCs. In terms of molecular mechanism, we further verified that EFL2 robustly downregulated TLR7 mediated IRAK4-IKKβ-IRF5 and NF-κB signaling pathways activation, and blocked IRF5 and p65 phosphorylation and translocation activity. Taken together, our data indicate EFL2’s therapeutic potential as a candidate for rheumatoid arthritis and other TLR7-dependent diseases.

## Introduction

Rheumatoid arthritis (RA) is a chronic inflammatory autoimmune disease characterized with synovitis, cartilage erosion and joints damage (Orr et al., 2018). Although the precise etilogy of RA is still unclear, the acknowledged critical step in the process of RA initiation and development is the immune break including the innate and adaptive immune cells activation, followed by excessive pro-inflammatory cytokines, chemokines and different antibodies production (Orr et al., 2018). The hallmark of RA is the chronic synovitis associated with massive amounts of innate immune cells infiltration (e.g., mast cells, neutrophils, monocytes, dentritic cells, and macrophages) as well as small proportions of T and B lymphocytes (Orr et al., 2018). Antigen-activated CD4+T cells can stimulate myeloid cells, especially neutrophils and macrophages. These activated cells then interact with synovial fibroblasts, osteoclasts and chondrocytes to release matrix metalloproteinases (MMPs), RANK ligand (RANKL), cyclooxygenase 2 (Cox2), and prostaglandins generation which contribute to sustained synovitis, cartilage erosion, and joint damage (Choy, 2012; Lee et al., 2013; You et al., 2014).

TNF-α and IL-1β, the well-known cytokines which play a critical role in etiology of RA, are produced mainly by monocytes and macrophages. TNF-α receptors are expressed on a variety of target cells. Their activation can trigger production of other cytokines, induce endothelial adhesion molecules, and stimulates collagenase and osteoclast differentiation (Weinblatt, 1992; Lee et al., 2013). Therefore, TNF-α was previously investigated as a target in RA treatment (Yamanaka, 2015). IL-1β displays activity in RA that is similar to TNF-α. They activate intra-cellular signal-transduction pathways that go through a series of kinases, leading to the activation of nuclear-factor kappa B (NF-κB) (Weinblatt, 1992). Previous reports have showed that blockage of IL-1 was significantly effective in RA, reducing inflammation, bone destruction and disease progression (Joosten et al., 1999; Pascual et al., 2005). IL-6 is another pro-inflammatory cytokine produced by T cells, monocytes, macrophages, and synovial fibroblasts (Hunter and Jones, 2015). It is reported that RA patients have elevated serum levels of both IL-6 and IL-6R in serum and synovial fluid (Santos Savio et al., 2015). IL-6 has a crucial role in the inflammatory processes once at the joint, including osteoclast-mediated bone resorption and pannus development (Srirangan and Choy, 2010). In the treatment of RA, IL-6 blockade has proven to be very useful for those patients who are refractory to conventional therapy or TNF inhibitors (Narazaki et al., 2017). Chemokines also play an important role in the recruitment of leucocyte subset in inflamed joints in RA. Blocking CXC chemokine receptor 3(CXCR3) or C-C chemokine receptor 5 (CCR5) have the potent anti-arthritic effects on collagen-induced arthritis (CIA) in DBA/1J mice, which was mainly due to the NF-κB expression suppression in immune cells (Bakheet et al., 2020; Ansari et al., 2021). Due to the importance of inflammatory cytokines/chemokines in RA pathogenesis, targeting the key cytokines or upstream signaling activation will be useful strategy to control clinic symptoms and alleviate disease severity.

Toll-like receptors (TLRs), a family of innate pattern recognition receptors, are widely involved in the regulation of innate immunity. So far, 10 TLRs substypes have been identified (Kawai and Akira, 2010). Among TLRs, the impact of TLR 2, 3, 4, seven and 9 on arthritis development has been investigated in STA model in details. TLR2 exhibits an inhibitory role in STA model by controlling the inhibitory FcrRIIB receptor on macrophages (Abdollahi-Roodsaz et al., 2013). TLR9 activation facilitates the binding of unmethylated DNA CpG motifs (CpGs) to dentritic cells (DCs) and the subsequent crosstalk with natural kill cells (NKs), leading to a significant reduction of neutrophil migration into the joint (Wu et al., 2007). Though TLR4 is normally thought to play a pathogenic role in diseases, the impacts of TLR4 on the progression of arthritis in STA model are inconsistent in different studies (Choe et al., 2003; Kim and Chung, 2012). On the contrast, it is clear that TLR3 and seven play a pivotal role in arthritis progression. Moreover, they also have a synergy effect on IL-1β production, which is mostly dependent on the interferon regulatory factor (IRF5) function (Duffau et al., 2015). In addition, NF-κB signaling pathways, involved into inflammatory responses in the pathogenesis of RA (Bondeson et al., 1999; Tak et al., 2001), can also be activated in myeloid cells when TLR7/8 agonists bind to their receptors, and the subsequent cytokines production are cell-type dependent (Eng et al., 2018).

IRF5 is known as a key transcription factor involved in the control of the expression of pro-inflammatory cytokines responses to microbial infection and type I interferon responses to virus (Krausgruber et al., 2010). Recently, polymorphisms in the IRF5 gene have been found to associate with an increased risk of developing rheumatoid arthritis, lupus erythematosus and inflammatory bowel diseases (Demirci et al., 2007; Balasa et al., 2010; Dawidowicz et al., 2011). As a central transcription factor of TLR7 signaling, IRF5 can affect neutrophil influx to the inflammatioin sites, define the inflammatory macrophages phenotype and impact cytokines production (Schoenemeyer et al., 2005; Weiss et al., 2015). TLR7 induced IL-1β production in synovial fibroblasts and M1-type macrophage from rheumatoid arthritis patients was also in an IRF5-dependent manner (Guo et al., 2018). In addition, IRF5 also has the species-invariant role in controlling the inflammatory macrophage phenotype (Weiss et al., 2013). Depletion of IRF5 is able to protected mice from methylated BSA-induced acute arthritis or K/BxN serum transfer arthritis, suggesting that IRF5 could be an attractive therapeutic target in arthritis.

*Euphorbia L*. are traditional medicine for folk medicine practice (Li et al., 2003)**.** They are reported to have many pharmacological effects and be used in the treatment of ascites syndrome, abscess, arthralgia syndrome and hemorrhage syndrome (Li et al., 2003). A previous study reported that supplementation with *Euphorbia hirta* (*E. hirta*) extract significantly alleviated the adjuvant induced arthritis (AIA) in mice by decreasing the levels of inflammatory-mediators (Ahmad et al., 2014). The seeds of *Euphorbia lathyris L*. are the most typical and traditionally used herbs. Due to the mupltiple pharmacological effects, they have been added in chinese herbal compound preparation such as Zijingding and the traditional Chinese medicine prescription “Tongguansan” (Mei, 1989; Fan and Zhang, 1991). In a series of latheyran editerpenes extracted from *Euphorbia lathyris L*. seeds, known as *Euphorbia* factor L1-L11 (EFL1-11), EFL2 has been reported to have anti-cancer ability. It induced the apoptosis of lung cancer cell line A549 in a dose-dependent manner *in vitro* (Lin et al., 2017). A separate study also indicated its inhibitory effect tumor growth by suppressing SMMC-7721 and Hep G2 cells proliferation and migration via STAT3 phosphorylation (Fan et al., 2019). Our recent study identified EFL2’s robust improving effect on LPS-induced lung injury in mice (Zhang et al., 2018). In the present study, we assessed the pharmaceutic and pharmacokinetic features of EFL2, and evaluated its protective effect on K/BxN serum-transfer arthritis in mice. Moreover, we determined its inhibitory role on TLR7 mediated signaling pathways and elucidated its anti-arthritis mechanisms both *in vivo* and *in vitro*.

## Materials and Methods

### Preparation of EFL2

**FIGURE 1:**
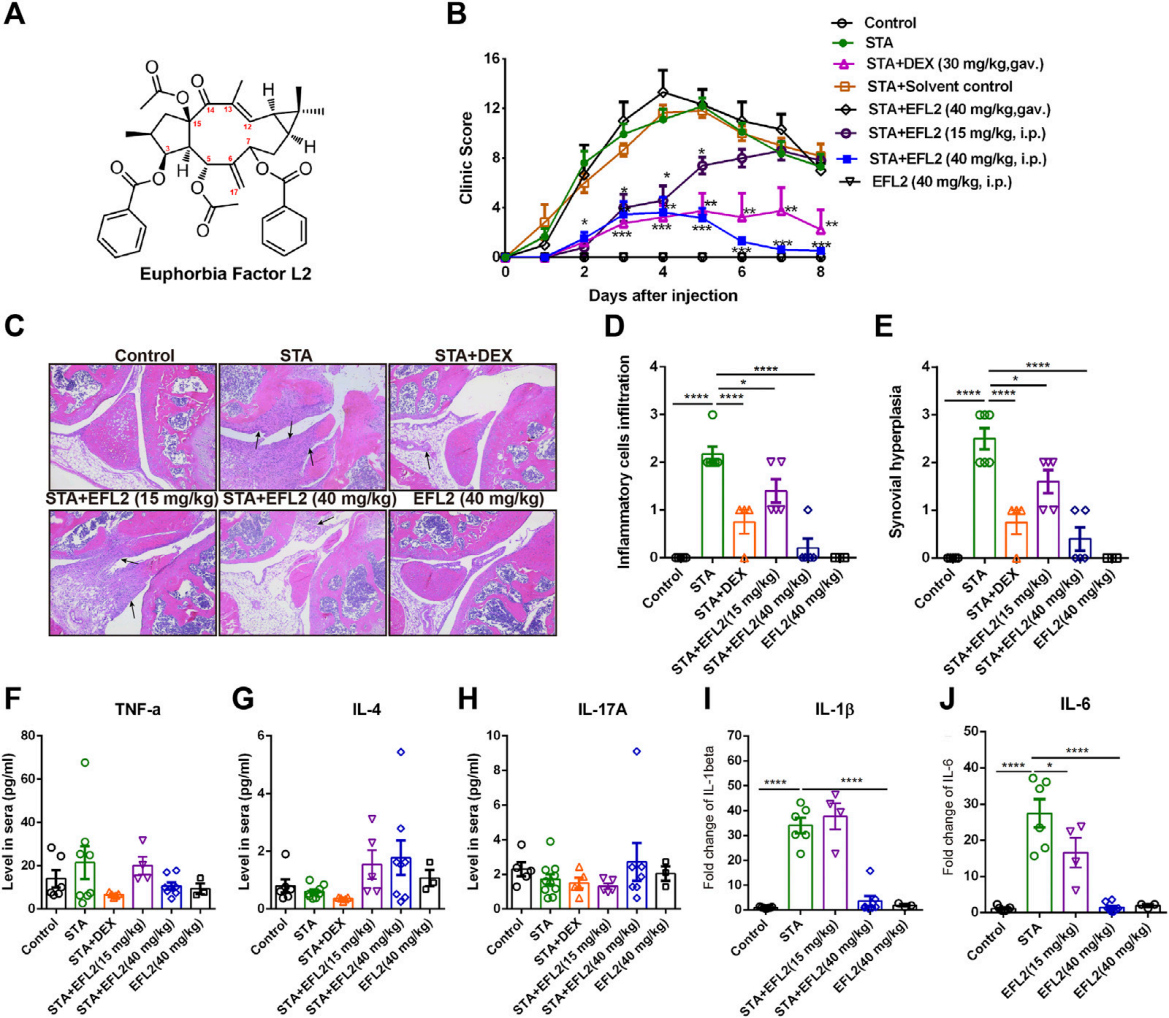
Euphorbia factor L2 effectively alleviates K/BxN serum transfer arthritis (STA) in mice **(A)** Chemical structure of EFL2 (C_38_H_42_O_9_, molecular weight = 642.735) **(B)** Arthritic index in each group was recorded every day after serum injection (day 0) **(C)** Representative hematoxylin and eosin (H.E.) staining images of the knee joints. Assessment of inflammatory cell infiltration **(D)** and synovial hyperplasia **(E)** in the knee joints from the control (n = 6), STA (n = 8), STA mice treated with DEX (n = 5) or EFL2 (15 or 40 mg/kg) (n = 5–8) and mice solely treated with EFL2 (40 mg/kg) (n = 3) **(F-J) **TNF-α **(F)**, IL-4 **(G)**, IL-17A **(H)**, IL-1β **(I)** and IL-6 **(J)** levels in the blood of control, STA and STA mice treated with DEX or EFL2. Data represent the mean values ±SEM. *p < 0.05, **p < 0.01, ***p < 0.001 were considered as significant.

Isolation and identification of EFL2 (C_38_H_42_O_9_, Figure 1A) were performed as described before (Zhang et al., 2018). The purity of up to over 98% was determined by HPLC. EFL2 was dissolved in DMSO as a stock solution. The stock solution was diluted (1:100) with sterile PBS supplemented with 3% tween 20 before administrated to mice (the final concentration of DMSO is 1%).

### Establishment of K/BxN Serum-Transfer Arthritis Model and Animal Treatment

K/BxN serum was collected from K/BxN mice at 8 weeks of age, pooled, aliquoted and stored at -20 °C. K/BxN serum-transfer arthritis (STA) model was induced by intraperitoneal (i.p.) application of 150 μL of serum at day 0. Clinical score for arthritis severity was assessed using a 0–4 point scale for each paw at various time points (0–16 total score) as described previously (Luo et al., 2011). 8-week old male C57BL/6 mice were purchased from Dashuo Animal Center (China, Chengdu), maintained at 25°C, and exposed to 12-h light and dark cycles. Mice were fed with standard laboratory chow and water with 1 week of acclimatization. Naive mice were used as the control group. Except the control mice, all mice were i. p. injected with 150 μL of serum, then randomly divided into the following groups and treated with different compounds daily for the consecutive 8 days: 1) STA group; 2) Solvent control group, mice were gavaged with the solvent; 3) Dexamethasone (DEX) group, mice were gavaged with 30 mg/kg DEX; 4) EFL2 group 1, mice were gavaged with 40 mg/kg EFL2; 5) EFL2 group 2, mice were intraperitoneally injected with 15 mg/kg EFL2; 6) EFL2 group 3, the control mice were intraperitoneally injected with 40 mg/kg EFL2. In addition, the control mice were intraperitoneally injected with 40 mg/kg EFL2. All animal experiments were conducted in accordance with ethical regulation for animal care and use in China.

### *In vivo* Pharmacokinetic Study

Prior to the pharmacokinetic study, 12 male C57BL/6 mice were fasted for 12 h with free access to water, and then treated by intraperitoneal injection of EFL2 at a dose of 40 mg/kg. About 50 μL blood samples were collected via the oculi chorioideae vein at 0.5, 1, 2, 4, 8, 12, 24, 72, 96, and 168 h, respectively. After centrifugation at 5,000 rpm for 10 min, plasma samples were obtained and frozen at −80°C until analysis. The pharmacokinetic parameters of EFL2 were calculated using WinNolin 4.0.1 software.

### Preparation of Plasma Sample

20 μL plasma samples were spiked with 340 μL acetonitrile by vortex mixing for 30 s. The mixture was then centrifuged at 10,000 rpm for 5 min at 4°C to separate the precipitated protein. An aliquot of 50 μL supernatant was transferred to a fresh tube and spiked with 450 μL mobile phase (acetonitrile containing 0.1% formic acid). After centrifugation at 13,000 rpm for 5 min, 3 μL supernatant was injected into Agilent 6,495 Triple Quadrupole LC/MS/MS system for each time. Then, the concentration of EFL2 was determined.

### Instrumentation and UPLC-MS Method

An Agilent 1,200 series UPLC system (Agilent Technologies, Waldbronn, Germany) equipped with an 6,495 quadruple mass spectrometer (Agilent Technologies, Santa Clara, CA, United States) were used for pharmacokinetic study. The separation was carried out with Waters Acquity UPLC BEH C18 column (2.1 × 50 mm, 1.7 μm) at a constant temperature of 30 °C. The mobile phase was composed of aqueous solution (0.1% formic acid, A) and acetonitrile (containing 0.1% formic acid, B) and the program of the mobile phase was as follows: 0 min, 40% B; 1 min, 40% B; 1.5 min, 80% B; 2.5 min, 90% B; 4 min, 90% B; 4.1 min, 40% B; 6 min, 40% B, at a flow rate of 0.3 ml/min. The mass spectra were acquired using a triple quadrupole mass spectrometer with ESI ion source in positive mode. The ionization conditions were as follows: drying gas temperature (N2), 300°C; gas flow, 11 L/min (N2); nebulizer pressure, 35 psi (N2). An Agilent Mass Hunter workstation was used for data processing.

### Histological Assessment

The left knee joints were removed on day 13 post STA and fixed in 4% formaldehyde overnight at 4°C. The tissues were decalcified in EDTA and sectioned after embedding in paraffin. 5 μm sections were deparaffinized with xylene, re-hydrated with 100, 95 and 80% ethanol and then stained with hematoxylin and eosin (H&E). Histopathological changes in the knee joints were measured using a semiquantitative scoring system (0–4 scale) to assess synovial hyperplasia and articular inflammatory cells infiltration as previously described (Yamanishi et al., 2002).

### Flow Cytometry Analysis

150 μL peripheral blood and the hind ankles were collected on day 5 and 13 post KBx/N serum transfer, respectively. For the peripheral blood samples, red blood cells were lysed and cells were prepared as single cell solution. The hind ankles were cut into 1 mm^2^ pieces, and then digested in 10% DMEM containing Collagen IV (1 mg/ml) for 1 h at 37°C. Single cell solution was then prepared after passing through 40 μm cell strainer. 1×10^6^ cells were stained with FITC-conjugated CD11b (BD, United States, 1:500), PE-conjugated F4/80 (BD, United States, 1:400), PerCP-Cy5.5-conjugated Ly6G (BD, United States, 1:400) and APC-conjugated Ly6C (BD, United States, 1:200) to detect macrophage, neutrophil and monocyte. Data were acquired using a BD FACSCalibur flow cytometer (BD Biosciences) and were analyzed by using FlowJo software (Version 7.6.1).

### Cell Preparation and Culture

RAW264.7 cells, a mouse macrophage cell line, were cultured in DMEM medium containing 10% fetal bovine serum (FBS), 1% penicillin-streptomycin and maintained at 37 °C in 5% CO_2_ humidified air. Bone marrow-derived macrophages (BMDMs) generation was performed as following description. In brief, bone marrow cells were harvested from tibias and femurs of C57BL/6 mice and differentiated for 7 days in DMEM medium supplemented with 10% FBS, 1% penicillin-streptomycin and 50 ng/ml M-CSF. For human peripheral blood mononuclear cells (PBMCs) preparation, 5 ml peripheral venous blood was obtained from healthy donors in West China Hospital. PBMCs were isolated with Human Lymphocyte Separation Medium (LTS1077, Tian Jing, China) according to the manufacturer’s instructions. The experimental protocols were performed according to the approved guidelines established by the Institutional Human Research Subject Protection Committee of the Ethics Committee of West China Hospital, Sichuan University, China.

### Cell Viability Measurement

The effects of EFL2 on RAW264.7 cells, BMDMs and PBMCs viability were evaluated by Cell Counting Kit-8 (CCK8) assay (Dojindo, Japan). Briefly, RAW264.7 cells (1×10^5^ cells/well), BMDMs (1×10^5^ cells/well) and PBMCs (1×10^6^ cells/well) were plated into 96-well plates and then incubated at 37 °C in 5% CO_2_ incubator overnight. The medium was disposed next day and cells were incubated with different concentrations of EFL2 (0.1, 0.5, 1, 5, 10, 25, 50 and 100 µM) for 20 h. After disposing the supernatant, 100 μL of culture medium supplemented with 10 μL of CCK8 were added into wells and incubated for an additional 4 h. The optical absorbance at 450 nm was read with a Biotek Eon™ microplate reader (Biotek, Vermont, United States).

### ELISA Assay for Cytokines Measurement

RAW264.7 cells (5×10^5^/ml), BMDMs (5×10^5^/ml) and PBMCs (2×10^6^/ml) were plated in 96-well plates and incubated overnight. RAW264.7 cells (5×10^5^/ml) or BMDMs were either solely stimulated by poly:IC (1 μg/ml) (InvivoGen, United States), R837 (20 μg/ml) (Invegen, United States) and the combination of poly:IC and R837, or pretreated with EFL2 (1, 5 and 10 µM) 30 min prior to poly:IC and/or R837 stimulation for 24 h. For the anti-inflammatory molecular mechanism study, RAW264.7 cells (5×10^5^/ml) were plated in 96-well plates and stimulated with R837 (20 μg/ml) with or without the addition of NF-κB inhibitor (JSH-23, 10 μM/ml) for 24 h. After 24 h, the culture supernatant was collected for cytokines measurements.

IL-4, IL-17A and TNF-α in sera from different group mice were measured using CBA Multiplex kit (BD, United States). The concentrations of IL-1β and IL-6 in serum or supernatant were detected by ELISA Kit (MULTI SCIENCES, China) according to the manufacturers’ instructions.

### Western Blot Assay

RAW264.7 cells (2×10^6^ cells/well) were plated into 6-well plates and then incubated at 37°C in 5% CO_2_ for 24 h. After incubated with various concentrations of EFL2 (1, 5 and 10 µM) for 1 h, cells were then stimulated by R837 (20 μg/ml) for 30 min. Cell lysates were prepared by direct lysis in 20 µL IP buffer (Beyotime Biotechnology, Shanghai, China). After obtaining the whole cell extracts, the cytosolic and nuclear were seperated using Nuclear and Cytoplasmic Protein Extraction Kit (Beyotime Biotechnology, Shanghai, China). BCA Protein Assay Kit (Beyotime Biotechnology, Nantong, China) was applied to determine the protein concentrations. The bands corresponding to p- IKKα/β, IKKα/β, *p*-IκBα, IκBα, p-p65, p65, *p*-IRAK4, IRAK4, *p*-IKKβ, IKKβ, *p*-IRF5, IRF5, β-actin and Lamin B were visualized using a Western Blot Detection Chemiluminescence Kit (Merck). Protein extracted from the total lysates/cytoplasm and nucleus was quantified in relation to β-actin and Lamin B bands, respectively.

The antibodies used in the experiments were as follows: rabbit anti-mouse IKKα/β polyclonal antibody (1:1,000, Abways, China), rabbit anti-mouse IκBα polyclonal antibody (1:1,000, Abgent, China), rabbit anti-mouse p65 polyclonal antibody (1:1,000, Abgent, China), rabbit anti-mouse phosphorylated IKKα (Ser176)/β (Ser177) (1:1,000, Abways, China), rabbit anti-mouse phosphorylated IκBα (1:1,000, Abcam, United States), rabbit anti-mouse phosphorylated p65 (1:1,000, Abcam, United States), rabbit anti-mouse IRAK4 polyclonal antibody (1:1,000, Proteintech, United States), rabbit anti-mouse phosphorylated IRAK4 (Thr345/Ser346) (1:1,000, Affinity, United States), rabbit anti-mouse IKKβ polyclonal antibody (1:1,000, Abbkine, United States), rabbit anti-mouse phosphorylated IKKβ (Ser177) (1:1,000, Abways, China), rabbit anti-mouse IRF5 polyclonal antibody (1:1,000, Immunoway, United States), rabbit anti-mouse phosphorylated IRF5 (Ser437) (1:1,000, Affinity, United States), anti-mouse actin monoclonal antibody (1:20,000, Transgen, China) and rabbit anti-mouse LaminB monoclonal antibody (1:5,000, Abways, China).

### Transfection of IRF5 Plasmid

RAW264.7 cells (4×10^5^ cells/well) were seeded into 6-well plates before transfection with the plasmid. When cells confluency reached 60%, RAW264.7 cells were transfected with plasmid IRF5-mouse vector (Origene, United States) using TransEasy™ Transfection Reagent (FOREGENE, China). IRF5 plasmid was suspended with transfection reagent as 1:1.5. Cells were incubated at 37°C for 24 h, then treated with EFL2 and R837 for another 24 h. The supernatant was collected for IL-1β and IL-6 measurements using ELISA kits as described above. For western blot detection, RAW264.7 cells were pretreated with EFL2 (10 µM) for 1 h and stimulated with R837 (20 μg/ml) for 40 min after IRF5 plasmid transfection. Cell lysates were prepared as described above.

### Confocal Microscopy

P65 and IRF5 translocation activity was analyzed by confocal microscopy. Briefly, 1×10^5^ RAW264.7 cells were plated on coverslips overnight. Cells were pretreated with various concentrations of EFL2 (1, 5 and 10 µM) for 1 h and then stimulated by R837 (20 μg/ml) for 30 min. After washed with ice-cold PBS, RAW264.7 cells were fixed with 4% paraformaldehyde for 20 min at room temperature. The coverslips were washed with PBS with 0.2% tween 20 and blocked with 5% goat serum. Primary antibodies were applied to coverslips overnight at 4°C, followed by secondary antibodies incubation for 1 h at room temperature. After three times wash, coverslips were mounted with Fluoroshield™ with DAPI (Solarbio, China) and images were acquired by confocal microscopy (ZEISS, Germany). Primary antibody: rabbit anti-mouse IRF5 (1:800, Abcam, China); rabbit anti-mouse p65 (1:1,000, Abcam, China). Secondary antibody: Alexa eFluor^®^488 conjugated goat anti-rabbit IgG (1:2000, Thermo Fisher Scientific, Carlsbad, United States).

### Immunochemistry Staining

The dissected left ankles were fixed in 4% formaldehyde overnight. Embedding, de-paraffinization, and re-hydration of the ankle sections were the same as described for H&E staining. After treated with 3% hydrogen peroxidase for 10 min, the tissue sections were immersed in a 10 mM sodium citrate buffer (pH 6.0) for 15 min at 95°C, and washed with PBS. The sections were then blocked with 5% goat serum in PBS, and incubated with primary antibodies overnight at 4°C. Primary antibodies were as follows: rabbit anti-mouse IL-1β (1:600, Abcam, China); rabbit anti-mouse IL-6 (1:50, Abcam, China). The signal was visualized using an Envision System (DAKO, United States). The images were then acquired by microscopy (Leica DMil).

### PCR Array Analysis

Total RNA was isolated from the right ankles using the TRIzol Reagent (Invitrogen) and purified with RNeasy Mini Kit and RNase-Free DNase Set (Qiagen). RNA quality was determined using a spectrophotometer and was reversely transcribed using Wcgene^®^ mRNA cDNA kit. The complementary DNA was used on the Toll-like Receptor Pathway PCR Array plate (Wcgene^®^ biotech, China).

### Quantitative RT-PCR

Frozen right ankles were pulverized with liquid nitrogen and total RNA was extracted using Trizol reagent (Invitrogen). RNA was reverse transcribed into cDNA using an oligo d(T) primer. The obtained cDNA was applied for quantitative RT-PCR using SYBR Green I-dTTP (Eurogentec). Samples were analyzed in triplicate, and β-actin was used as endogenous controls. Data was analyzed using the 2^−ΔΔCt^ method and is expressed as “Fold change” (expression relative to housekeeping gene and normalized to reference sample).

### Statistical Analysis

Data are presented as the mean ± SEM. Statistical analysis for multiple comparisons was performed by one-way ANOVA followed by Bonferroni’s post-hoc comparisons test using GraphPad Prism 8.0 software. *p < 0.05, **p < 0.01, ***p < 0.001, ****p < 0.0001 were considered as significant difference.

## Results

### EFL2 Effectively Alleviates Arthritis Severity in STA Mice

Developing anti-arthritic drugs and verifying therapeutic effect can be achieved by taking advantage of animal arthritis models. Although many murine models of arthritis have inflammation effector features, the injection of K/BxN mice serum containing antibodies against glucose-6-phosphate isomerase (G6PI) mainly induce inflammatory arthritis by initiating innate immune system. Unlike collagen-induced arthritis, myeloid cells exert a central role in K/BxN serum-transfer model, but T and B cells are not required (Christensen et al., 2016). Macrophages, neutrophils and Ly6C^−^ nonclassical monocytes are major pivotal cell types which play an essential role in the initiation, progression and resolution of sterile joint inflammation in K/BxN serum-transfer arthritis (STA) model (Solomon et al., 2005; Misharin et al., 2014).

In this study, we evaluate the inhibitory effect of EFL2 on inflammatory arthritis, and also test the therapeutic efficiency of EFL2’s gavage and intraperitoneal (i.p.) injection in STA mice. The clinic scores in STA mice showed a rapidly increased clinic score following with slow remission from day 5–8 (Figure 1B). Meanwhile, histological assessment of the joints demonstrated abundant infiltrated inflammatory cells in the knee of STA mice, accompanied with obvious synovial hyperplasia (Figures 1C–E).

In contrast, DEX administration significantly lowered arthritis clinical scores, and reduced infiltrated cells in the joints of STA mice (Figures 1B–E). In EFL2-treated groups, EFL2 oral administration did not affect the arthritis severity of STA mice at all (Figure 1B). Whereas, i. p. injection with the same dose of EFL2 (40 mg/kg) dramatically improved joint swelling and redness in STA mice (Figure 1B). 15 mg/kg of EFL2, to a moderate extent, alleviated arthritis symptoms, particularly in the acute phase of arthritis (Figure 1B). Mice were sacrificed on day 8 and there were abundant inflammatory cells infiltrating into the joint in STA mice, accompanied with obvious synovial hyperplasia (Figures 1C–E). As shown in Figure 1C, 15 mg/kg of EFL2 only partially reduced the excessive inflammatory cells infiltration and synovium hyperplasia in STA mice. In contrast, high dose of EFL2 (40 mg/kg) or DEX treatment significantly inhibited inflammatory status and inflamed synovium in joints of STA mice (Figures 1C–E).

Inflammatory cytokines (e.g. TNF-α, IL-17, IL-1β and IL-6) are known to be pathogenic factors triggering joint diseases and established synovitis (Martin et al., 2017). The impact of EFL2 on a series of pro-inflammatory cytokines production was also assessed. Interestingly, no significant changes in TNF-α, IL-4 and IL-17A levels were observed among different groups (Figures 1F–H). Unlike TNF-α, IL-1β is absolutely required for disease development in K/BxN serum transfer arthritis (Ji et al., 2002). Meanwhile, IL-6 is also considered as a key inflammatory mediator in the pathogenesis of rheumatoid arthritis (Calabrese and Rose-John, 2014). Over 2-fold levels of IL-1β and IL-6 were detected in the sera of STA mice, which were significantly reduced by EFL2 (40 mg/kg, i. p.) or DEX treatment (Figures 1I,J). In contrast, 15 mg/kg of EFL2 only effectively inhibited IL-6 level of STA mice on day 8 (Figure 1J). Taken together, above results indicate that the intraperitoneal administration of EFL2 is able to ameliorate the disease severity of STA, which might due to the decrease of pro-inflammatory cytokines production in serum.

### Pharmacokinetic Study of EFL2

An UPLC-MS/MS method was successfully developed and applied to a pharmacokinetic study of EFL2 in the plasma after intraperitoneal injection. The main pharmacokinetic parameters of EFL2 were shown in Table 1. After 40 mg/kg intraperitoneal injection, the concentration of EFL2 reached a maximum plasma concentration (C_max_) of 4,525.12 ± 1,630.01 ng/ml, and the time to reach the maximum concentration (T_max_) was 2.50 ± 1.73 h. The short T max indicated that EFL2 was rapidly absorbed *in vivo* and produced quick therapeutic effects. The area under the plasma concentration versus time curve from zero to time t (AUC_0–t_) was (374.10 ± 163.96) h·ng/ml for EFL2. Besides, EFL2’s half time (t_1/2_) value was 21.17 ± 7.11 h, which suggested that EFL2 was slowly eliminated *in vivo*.

**TABLE 1:** Pharmacokinetic parameters of ELF2 in mice after intraperitoneal injection of EFL2 (n = 6 for each time points).

Parameter	Unit	EFL2
T_max_	h	2.5 ± 1.73
C_max_	ng/ml	4,525.12 ± 1,630.01
*t*1/2	h	21.17 ± 7.11
AUC_0-t_	h*ng/ml	374.10 ± 163.96
AUC_0-∞_	h*ng/ml	379.43 ± 165.88
MRT_0-t_	H	59.16 ± 2.75
MRT_0-∞_	H	61.38 ± 3.70

### EFL2 Suppresses the Inflammatory Cells Infiltration and Activation in Ankles of STA Mice

**FIGURE 2:**
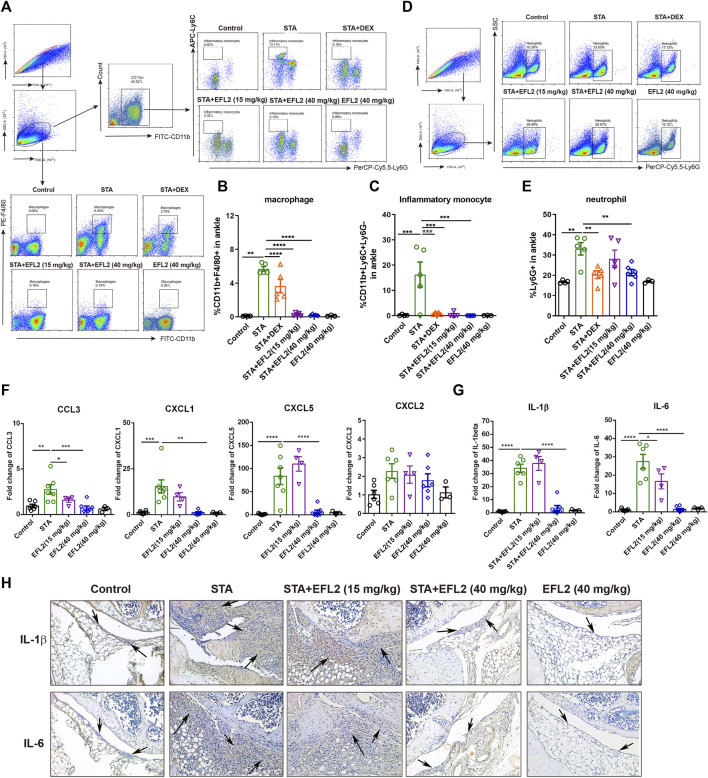
Euphorbia factor L2 suppresses inflammatory cells infiltration into the ankle. Flow cytometry gating strategy and quantification for macrophages (CD11b^+^F4/80^+^) **(A, B)**, inflammatory monocytes (CD11b^+^Ly6C^+^Ly6G^−^) (A, C) and neutrophils (Ly6G^+^) **(D, E)** in the ankles from the control, STA, STA mice treated with DEX or EFL2 (15 or 40 mg/kg) and mice solely treated with EFL2 (40 mg/kg) (n = 3-7 per group) **(G, H)** mRNA expressions of chemokine CCL3, CXCL-1, CXCL-5, CXCL2 **(G)** and inflammatory cytokine IL-1β and IL-6 **(H)** in the ankles from each group **(I)** Immunohistochemical staining for IL-1β and IL-6 in the ankle from the control, STA, STA mice treated with EFL2 (15 or 40 mg/kg) and mice solely treated with EFL2 (40 mg/kg). Arrows represent the synovium with numbers of infiltrated inflammatory cells. Data represent the mean values ±SEM. *p < 0.05, **p < 0.01, ***p < 0.001, ****p < 0.0001 were considered as significant.

The infiltration of myeloid cells, especially macrophages and neutrophils, is a prominent feature of synovium lesions, and is also positively correlated with the degree of joint erosion (Choy, 2012; Orr et al., 2018). Next, we performed flow cytometry to analyze different myeloid cells in blood and ankle of mice, respectively. Compared with control mice, STA mice showed dramatic increases in the proportions of neutrophil (Ly6G^+^) and inflammatory monocyte (CD11b^+^Ly6C^+^Ly6G^−^) in the circulation (Supplementary Figure S1). In addition, we detected predominant neutrophils infiltration in the ankles of STA mice (Figures 2D,E), accompanied with significantly increased percentages of macrophages and inflammatory monocytes (Figures 2A–C). 30 mg/kg DEX significantly inhibited the increases of these myeloid cells subsets in the ankles of STA mice (Figures 2A–E). While, EFL2, at the dose of 15 mg/kg, remarkably reduced macrophage and inflammatory monocyte influx into the ankles but not neutrophil (Figures 2B,C). 40 mg/kg of EFL2 treatment displayed a more robust suppressive effect on the expansions of these three myeloid cells subsets both in the blood and ankles of STA mice (Supplementary Figure S1, Figures 2A–E).

To assess whereby EFL2 administration prevents the infiltration of these myeloid cells in the ankles, we analyzed the mRNA expressions of known neutrophil chemoattractant, such as CXCL1, CXCL2, CXCL5, and chemokine (C-C motif) ligand CCL3, CCL4 and CCL5 in the ankles of mice. CCL4 and CCL5 mRNA expressions were undetectable in our study. No significantly changes were observed in CXCL2 gene expression among different groups (Figure 2F). In contrast, CXCL1 and CXCL5 mRNA expressions were significantly up-regulated in the ankles of STA mice on day 5, which were remarkably down-regulated by 40 mg/kg EFL2 i. p. treatment (Figure 2F). Meanwhile, this compound also significantly reduced the gene expression of CCL3, known as macrophage trafficking, in STA mice (Figure 2F). In contrast, EFL2, at the dose of 15 mg/kg, only significantly decreased chemoattractant CCL3 mRNA expression (Figure 2F).

Since the infiltration of myeloid cells contributes to increased inflammatory cytokines levels in inflamed joints, we next detected mRNA expression of inflammatory cytokines in the ankles. Compared with control mice, IL-1β and IL-6 mRNA expression increased to 35- and 27-fold in the ankles of STA mice, respectively, which were robustly inhibited by EFL2 i. p. treatment (Figure 2G). Immunohistochemical staining for IL-1β and IL-6 further confirmed EFL2 could inhibit these two cytokines production in the local inflamed joints (Figure 2H). Collectively, these data indicate that EFL2 i. p. administration is able to inhibit inflammatory cells infiltration by suppressing neutrophil and macrophage chemoattractants and suppress their activation to reduce local inflammatory cytokines production.

### The Suppressive Role of EFL2 on TLR Signaling Pathway in Ankles of STA Mice

**FIGURE 3:**
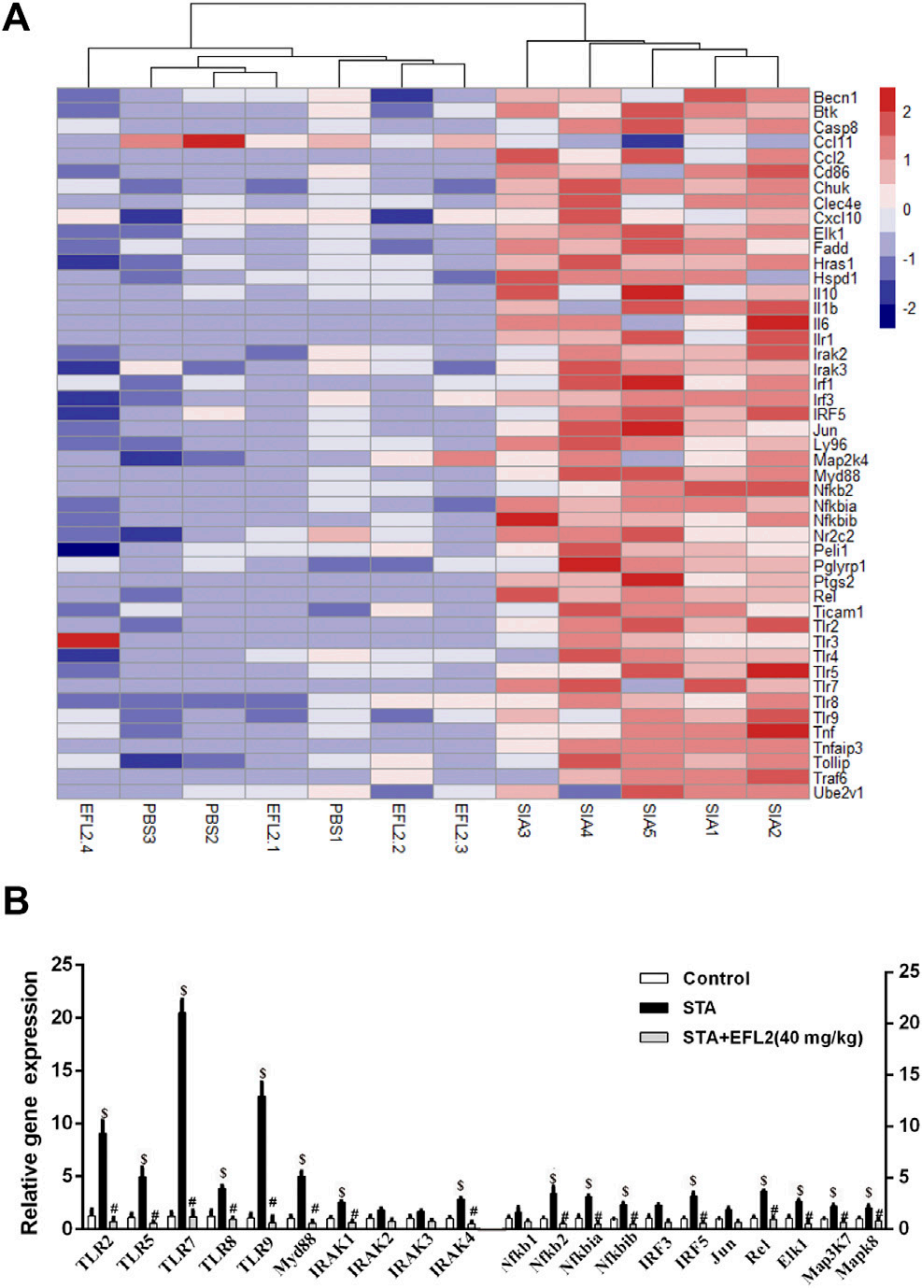
Euphorbia factor L2 suppresses TLR signaling pathway activation **(A)** Heat map showing the log(fold-changes) in TLR signalling pathway genome in the ankles of mice. Color scale ranges from red to blue which respectively denotes the up- or down-regulated of the genes **(B)** RT-PCR verified the changes in the transcriptional regulators mRNA expression among control, STA and STA mice i. p. injected with EFL2 (40 mg/kg) (n = 3 in control, n = 5 in STA and EFL2 treated groups). Data represent the mean values ±SEM. *p < 0.05, **p < 0.01, ***p < 0.001 were considered as significant. **$**: STA versus Control, *p* < 0.05; #: STA + EFL2 treatment versus STA, *p* < 0.05.

Toll-Like Receptors (TLRs) are reported to implicate into the initiation, progagation and remission phases of STA (Guo et al., 2018). To identify the most relevant TLR signaling pathway which is implicated into EFL2’s suppressive effect on STA and inflammatory cytokines production, PCR array was performed in this study. As shown in Figure 3A, 44 gene expressions were increased in ankle of STA mice compared with control mice. Recently, emerging newer data indicate the role of TLR7 in the pathogenicity of RA, particular in the aspect of rhematoid synovitis (Kim et al., 2016) and bone erosion (Kim et al., 2019). A recent study showed that TLR7 deficiency alleviated K/BxN serum-induced arthritis by imparing interferon regulatory factor 5 (IRF5) mediated IL-1β and IL-6 produced by macrophages and synovial fibroblasts, respectively (Duffau et al., 2015). We found a 16-fold increase in TLR7 expression at the transcriptional level in STA mice compared with control mice (Figures 3A,B). In addition, mRNA expressions of TLR2 and 9 which mediate an inhibitory role in STA model (Abdollahi-Roodsaz et al., 2013) were also significantly increased in STA mice. Consistently, STA mice displayed up-regulated mRNA expressions of other downstream genes including Myd88, IRAK1/4, Nfkbia/ib, IRF5, Rel, Map3K7 and Mapk8 (Figure 3B). While, 40 mg/kg EFL2 i. p. injection exerted a robust inhibitory effect on above up-regulated genes expression (Figure 3B). The inhibitory effects of TLR2 and TLR9 in STA pathogenesis can not explain the EFL2’s anti-inflammatory role in arthritis. Considering TLR7 mediated-downstream has a promoting role in inflammatory arthritis (Guo et al., 2018), we hypothesized that EFL2 may mainly interfere with TLR7-mediated signaling pathway in STA model to alleviate the inflammatory status in mice.

### EFL2 Strongly Inhibits TLR7-Induced Cytokine Production *in vitro*

**FIGURE 4:**
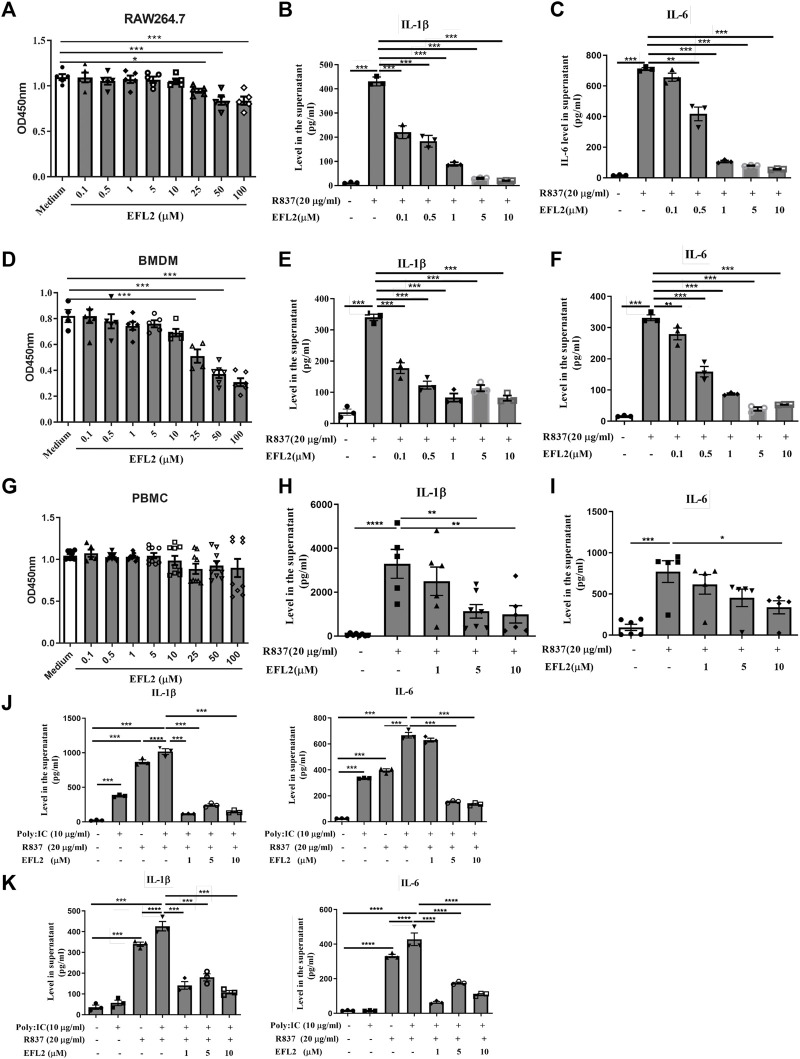
Euphorbia factor L2 reduces IL-1β and IL-6 production in R837-stimulated macrophages in vitro **(A, D, G)** Cytotoxicity effect of different concentrations of EFL2 (0.1, 0.5, 1, 5, 10, 25, 50 and 100 μM) on RAW264.7 cell **(A)** bone marrow-derived macrophages (BMDMs) **(D)** or PBMCs **(B, E, H)** Effect of different concentrations of EFL2 (0.1, 0.5, 1, 5, 10 μM) on IL-1β production in R837-stimulated RAW264.7, BMDMs and PBMCs **(C, F, I)** Effect of different concentrations of EFL2 on IL-6 production in R837-stimulated RAW264.7, BMDMs and PBMCs **(J, K)** The inhibitory effect of EFL2 on IL-1β and IL-6 secreted by RAW264.7**(J)** or BMDMs **(K)** under the co-stimulation of poly:IC and R837. n = 3 for each group in an independent experiment. Data represent the mean values ±SEM of three independent experiments. *p < 0.05, **p < 0.01, ***p < 0.001 were considered statistically significant.

EFL2 is a latheyran editerpene extracted from *Euphorbia lathyris L*. seeds with toxicity. We noticed that the peak concontration of EFL2 in sera was equal to 7.03 μM based on Cmax (4.52 ± 1.63 μg/ml) and its molecular weight (C_38_H_42_O_9_, MW = 642.6). Therefore, we set up the concentrations (from 0.1 to 100 μM) to test the toxicity of EFL2 on these cell lines. As shown in Figure 4. A and D, EFL2, did not display significant cytotoxicity in RAW264.7 cells or primary murine bone marrow-derived macrophages (BMDMs) until its concentration reached 25 μM (Figures 4A,D). In contrast, EFL2 did not affect PBMCs viability even at the high concentration of 100 μM (Figure 4G), suggesting that PBMCs are not sensitive to EFL2 treatment than murine macrophages.

Next, we test EFL2’s anti-inflammatory ability in murine macrophages and human PBMCs *in vitro*. R837, also known as imiquimod, is a strong TLR7 ligand agonist. It robustly induced IL-1β and IL-6 release in the supernatant of RAW264.7 cells, BMDMs and PBMCs (Figure 4). Notably, EFL2, even at the lower concentration of 0.1 μM, was able to effectively inhibit IL-1β secretion in RAW64.7 or BMDMs (Figures 4B,E). This compound also dose-dependently reduced IL-6 production in murine macrophages (Figures 4C,F). By comparison, the suppressive effect of EFL2 on IL-1β production was prior to that on IL-6 in human PBMCs (Figures 4B,C).

It is shown that TLR3 has a marked synergy role with TLR7 ligand for IL-1β production in BMDMs (Guo et al., 2018). In this study, we applied poly (I:C), a strong TLR3 agonist, to stimulate murine macrophages solely or together with R837. Poly (I:C) itself did not affect IL-1β and IL-6 production in BMDMs, but it significantly increased these two cytokines secretion in RAW264.7 cells. The addition of poly (I:C) did enhance IL-1β and IL-6 production in either R837-stimulated RAW264.7 cells or BMDMs (Figures 4J,K). Various concentrations of EFL2 to different expent decreased the levels of IL-1β and IL-6 in the supernatant of these two murine macrophages co-stimulated by poly (I:C) and R837 (Figures 4J,K). Overall these data demonstrate that EFL2 exerts effective anti-inflammatory role via inhibiting TLR7-induced proinflammatory cytokines production in murine macrophages.

### EFL2 Suppresses TLR7-Mediated IRAK4/IKKβ/IRF5 Signaling Pathway Activation

**FIGURE 5:**
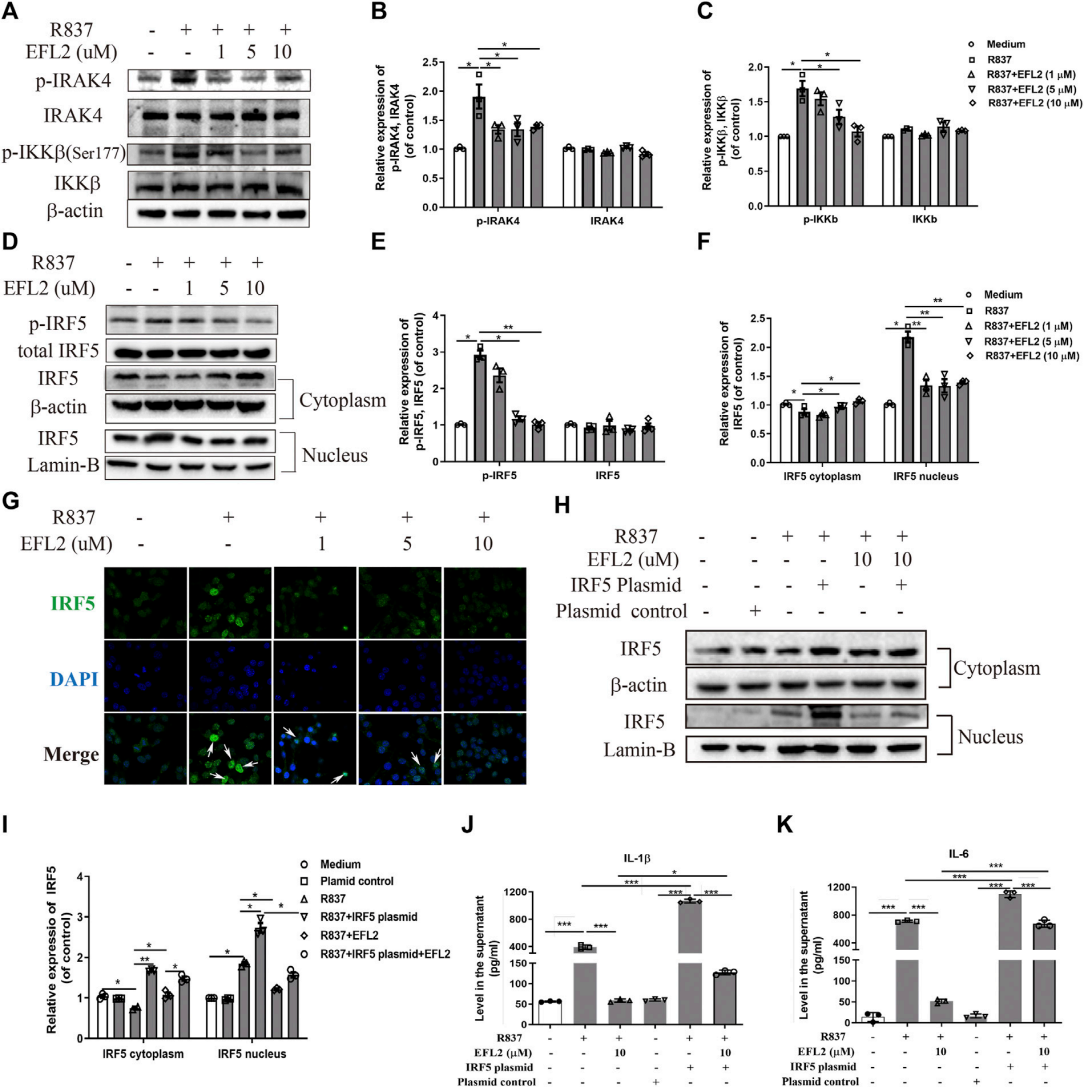
EFL2 reduced IL-1β and IL-6 production via robustly inhibiting TLR7-IKKβ-IRF5 signalling pathway **(A-C)** Western blot bands **(A)** and quantification of *p*-IRAK4, IRAK4 **(B)**, *p*-IKKβ and IKKβ expression **(C)** in RAW264.7 cells **(D-F)** Western blot bands **(D)** and effects of EFL2 on R837-induced IRF5 phosphorylation **(E)** and IRF5 expression in cytoplasm and nucleus **(F) (G)** IRF5 localization determined by immunofluorescence staining (Magnification: ×40) **(H)** IRF5 expression in cytoplasm and nucleus after IRF5 overexpression **(I)** Quantification of IRF5 expression after transfection **(J, K)** The levels of IL-1β **(J)** and IL-6 **(K)** produced by RAW264.7 cells before and after IRF5 overexpression (n = 3 for each group in an independent experiment). Data represent the mean values ±SEM of three independent experiments. *p < 0.05, **p < 0.01, ***p < 0.001 were considered as significant.

Following TLR7/8 activation, IRAK4 kinase can act with TAK1 to cause IKKβ phosphorylation, subsequently inducing IRF5 phosphorylation and translocation in human monocytes (Cushing et al., 2017). Based on above data, we hypothesize that TLR7-mediated IRF5 signaling pathway is implicated into the molecular mechanisms of EFL2’s anti-inflammatory effect. Similar to the previous findings in human monocyte (Cushing et al., 2017), R837 rapidly triggered the phosphorylation of IRAK4 and IKKβ in RAW264.7 cells (Figures 5A–C), followed with IRF5 phosphorylation at ser437 (Figures 5D,E). Additionally, R837 enhanced IRF5 translocational activity in 1 h, evidenced by the decreased expression in cytoplasm but increased expression in nucleus (Figures 5D,F). Immunofluorescence staining further confirmed that R837 dramatically induced IRF5 translocation from cytoplasm to nucleus (Figure 5G). While, EFL2 pretreatment significantly suppressed the phosphorylation of IRAK4, IKKβ and IRF5 in R837-stimulated RAW264.7 cells (Figures 5A–F). Meanwhile, IRF5 translocation was also effectively blocked by EFL2 at the concentrations of 5 and 10 μM (Figures 5D,F), which was also represented by less IRF5 positive staining in nucleus (Figure 5G).

To further confirm if the blockage of IRF5 activation is an essential process whereby EFL2 decreases pro-inflammatory cytokines reduction, IRF5 plasmid was transfected into RAW264.7 cells. As shown in Supplementary Figure S2, there was a significant upregulation of IRF5 expression in RAW264.7 transfected with IRF5, accompanied with IRF5 expression increase both in cytoplasm and nucleus (Figure 5H). Meanwhile, overexpression of IRF5 significantly reversed the suppressive effect of EFL2 on nuclear translocation of IRF5 (Figures 5H,I). Moreover, EFL2-induced reduction of IL-1β and IL-6 was effectively reversed with overexpression of IRF5 (Figures 5J,K). Overall, above data demonstrate that EFL2 is able to robustly block IRAK4-IKKβ-IRF5 signaling pathway activation, leading to pro-inflammatory cytokines IL-1β and IL-6 reduction.

### NF-κB Signaling Pathway is Implicated into EFL2 Caused Pro-Inflammatory Cytokines Reduction

**FIGURE 6:**
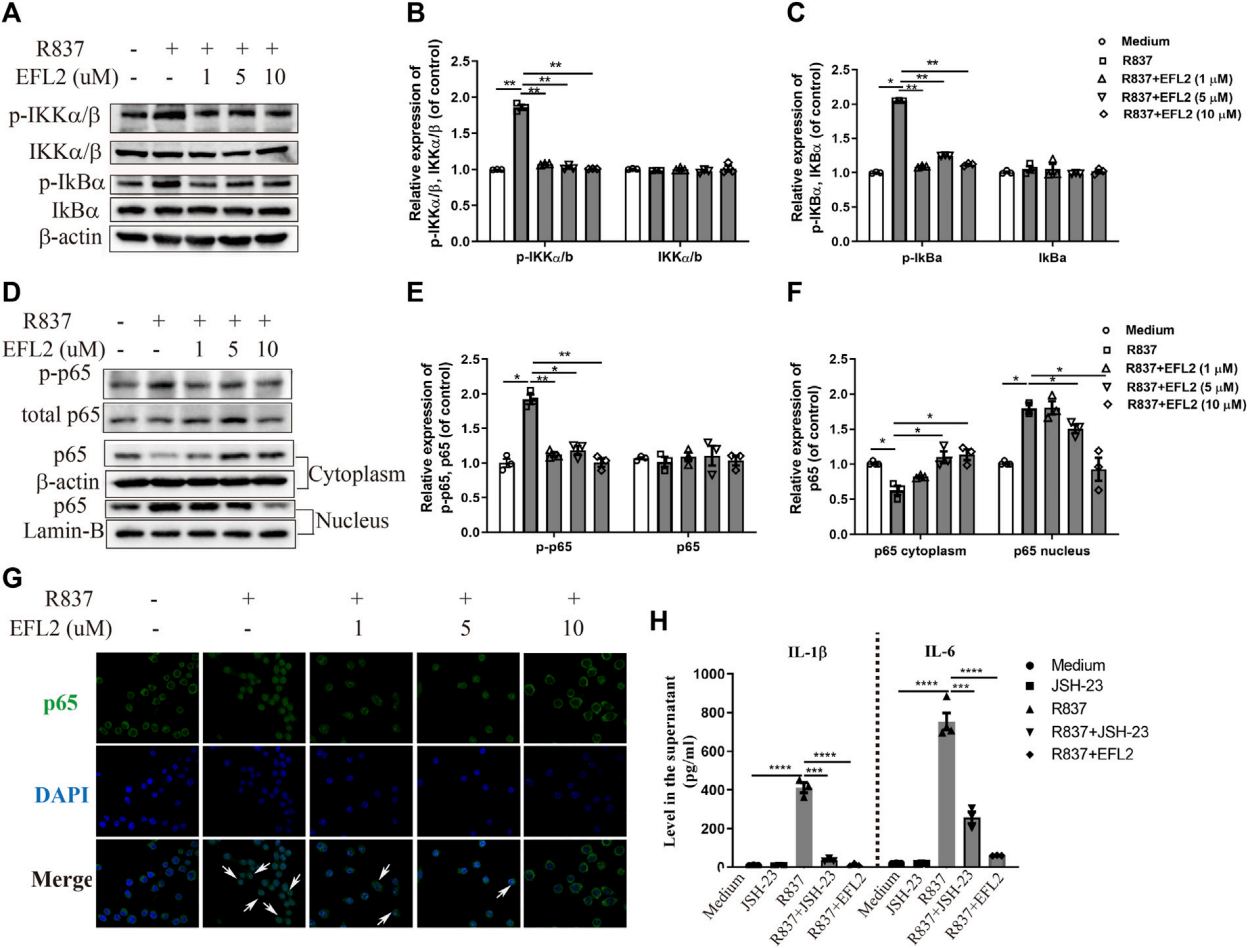
EFL2 inhibited the NF-κB signaling pathway activation. RAW264.7 cells were incubated with different concentrations of EFL2 (1, 5 and 10 μM) for 1 h, followed by R837 (20 μg/ml) stimulation for 30 min, then harvested for western blotting analysis **(A–C)** Western blot bands **(A)** and quantification of *p*-IKKα/β, IKKα/β **(B)**, *p*-IkBα and IkBα **(C)** in RAW 264.7 cells **(D–F)** Western blot bands **(D)** and quantification of p-p65 **(E)** and p65 expression in cytoplasm and nucleus **(F)** in RAW 264.7 cells **(G)** p65 localization determined by immunofluorescence staining (Magnification: ×40) **(H)** Effect of NF-κB inhibitor on R837-induced IL-1β and IL-6 production (n = 3 for each group in an independent experiment). Data represent the mean values ±SEM of three independent experiments. *p < 0.05, **p < 0.01, ***p < 0.01, ***p < 0.01 were considered as significant.

TLR7 agonist is reported to induce NF-κB activation in myeloid cells (Eng et al., 2018). Considering that IRF5 plasmid transfection did not completely reverse the reduced IL-1β production, we detected the impact of EFL2 on NF-κB activation in R837-stimulated RAW264.7 cells. As shown in Figure 6A, the phosphorylation of IKKα/β and IκB-α rapidly occurs in R837-stimulated RAW264.7 cells (Figures 6A–C), followed by NF-κB subunit p65 phosphorylation and translocation from cytoplasm to nucleus (Figures 6D–G). In TLR7-mediated NF-κB signaling activation, EFL2 effectively inhibited IKKα/β, IκB-α and p65 phosphorylation (Figures 6A,D). In addition, NF-κB subunit p65 translocation activity was also significantly suppressed by this compound (Figures 6D,G), which was further represented by less p65 positive staining in nucleus (Figure 6G). To further confirm if EFL2-induced IL-1β and IL-6 reduction was also NF-κB-dependent in our cell system, we test the effect of JSH-23, a NF-κB inhibitor, on these two cytokines production. Interestingly, JSH-23, to a large extent, blocked R837-induced IL-1β production but only partially reduced IL-6 secretion in RAW 264.7 cells (Figure 6H), suggesting that NF-kB activation was indeed involved into TLR7 activation induced-IL-1β and IL-6 secretion in RAW264.7 cells. Meanwhile, we noticed that EFL2, at the 10 μM, showed a stronger inhibitory ability on IL-1β and IL-6 production compared to JSH-23 (Figure 6H), suggesting NF-kB signaling pathway activation partially contributes to EFL2’s inhibitory effect on inflammatory cytokines release in macrophages.

## Discussion

Although etiopathology of RA is not fully understood, the central roles of TLRs in RA pathogenesis have been gradually uncovered in preclinical models. In this study, we identified a strong anti-arthritic effect of EFL2 in K/BxN serum transfer arthritis, where myeloid cells infiltration and inflammatory cytokines levels were significantly suppressed by EFL2. In addition, the molecular mechnism study explored that EFL2 exerted anti-IL-1β and IL-6 production role by suppressing TLR7-induced IRAK4-IKKβ-IRF5 and NF-κB signaling pathway activation.

In the current study, one interesting finding is the therapeutic difference of EFL2 on STA between gavage and intraperitoneal administration. Unlike i. p. administration, treatment of STA mice with equal dose of EFL2 by gavage hardly improved ankle redness or swelling in mice. There are many potential functional groups in the structure of EFL2, such as α, β-unsaturated ketone (at 14C position), terminal alkene (at 6C position) and four ester groups (two acetate groups at 5C and 15C position, two benzoate groups at 3C and 7C position). We speculate that the four ester groups are less stable than other functional groups in gastrointestinal environment because the pH in the gastrointestinal tract is beneficial for hydrolyzation of ester, and the different types of protease may accelerate this trend. This hydrolysis degradation of EFL2 is probably the crucial reason for the difference of pharmacological activity between the oral and intraperitoneal injection. Another reason which may lead to the low oral activity is that too many ester groups reduce the solubility of EFL2 and make it difficult to oral absorption.

Unlike TNF-α, IL-1β is absolutely required for disease development in K/BxN serum transfer arthritis (Ji et al., 2002). Although one study indicated that IL-6 did not play a major role in the inflammatory response in this model (Ji et al., 2002), it is still considered as a key inflammatory mediator in the pathogenesis of rheumatoid arthritis (Narazaki et al., 2017). In the measurement of a series of cytokines, we observed that EFL2 significantly reduced the levels of IL-1β and IL-6 in serum, but surpringly did not affect other cytokines. Given the pathologic manifestations in the K/BxN serum-transfer model are joint-specific (Ji et al., 2002), we also determined the effect of EFL2 on pro-inflammatory cytokines gene expression in the inflamed ankles. Similar to the trend in the circulation, IL-1β and IL-6 gene expression in STA mice was also significantly reduced by EFL2 treatment. One explanation is due to the EFL2-depended decrease in the levels of these two cytokines in the circulation. Another possibility is the reduction of infiltrated myeloid cells which are the important resources of IL-1β and IL-6 production in the ankles of STA mice.

Among TLRs, the implication of TLR2 and 4 in RA pathogenesis gained most attention in the earlier studies (Radstake et al., 2004; Huang et al., 2007; Davis et al., 2015). Recently, emerging newer data indicate the role of TLR7 in the pathogenicity of RA, particular in the aspect of rhematoid synovitis (Kim et al., 2016) and bone erosion (Kim et al., 2019). Potential TLR7 ligands, a GUUGUGU rich miR-Let7b, is found in human synovial fluid from RA patients (Kim et al., 2016). miR-Let7b can drive RA naive myeloid cell into M1 macrophages and promote inflammatory response in myeloid cells through TLR7 ligation (Kim et al., 2016). Besides the evidence in RA patients, TLR7 is also reported to implicate in the pathogenicity of both collagen-induced arthritis and STA model (Alzabin et al., 2012; Chen et al., 2012). Based on these reports and our PCR array data, we speculate EFL2 may inhibit the inflammaotry status in STA mice by interferring with TLR7 mediated-signaling pathway activation. Although TLR2 and TLR9 mRNA expressions were also significantly altered among the control, STA and EFL2 treatment groups (Figure 3B), the changes of TLR2 and TLR9 mRNA expressions can not explain the anti-inflammatory role of EFL2 in STA model because the inhibitory effects of TLR2 and TLR9 on STA (Abdollahi-Roodsaz et al., 2013). In fact, our *in vitro* experiments confirmed that EFL2 did have strong inhibitory effect on TLR7 activation-mediated inflammatory cytokines release in macrophages. Interestingly, in this study, we observed the dually inhibitory ability of EFL2 on TLR7-mediated NF-κB and IRAK4-IKKβ-IRF5 signaling pathway activation *in vitro*.

The transcription factor NF-κB plays a critical role in inflammation, cell proliferation, and apoptosis (Baeuerle and Baltimore, 1996; Vishva and Mak, 2002). Once the cell is stimulated by agents, the IκB is phosphorylated by IκB kinase (IKK) complex containning catalytic subunits IKKα and IKKβ(Mercurio et al., 1997; Ghosh and Karin, 2002). This phosphorylation targets IκB for degradation via the ubiquitin-proteasome pathway and allows nuclear translocation of classical NF-κB complexes, mostly p65 and p50 (Brown et al., 1995; Chen et al., 1995). Following TLR7/8 activation, a recent study uncovers that IRAK4 kinase acts with TAK1 to phosphorylate IKKβ, which subsequently induces IRF5 phosphorylation and translocation in human monocytes (Cushing et al., 2017). Importantly, IRAK4-IKKβ−IRF5 axis is independent of NF-κB (Cushing et al., 2017). IRF5 activation, involved in TLR7 downstream signaling pathways, can mediate the induction of pro-inflammatory cytokines down stream of TLRs, affect neutrophil influx to the inflammatioin sites (Weiss et al., 2015), and define the inflammatory macrophages phenotype and effective Th1 and Th17 cells generation (Krausgruber et al., 2011). A recent study also showed that TLR7 deficiency led to K/BxN serum-induced arthritis reduction by imparing interferon regulatory factor 5 (IRF5) mediated IL-1β and IL-6 generation in macrophages and synovial fibroblasts, respectively. Therefore, it is possible that EFL2 may exhibit anti-inflammation effect by suppressing these two signaling pathways. The findings that the phosphorylation of IKKα/β, an upstream activator, was significantly inhibited by EFL2 supported the above hypothesis. On the other hand, the production of IL-1β in RAW264.7 cells was only partially rescued after IRF5 plasmid transfection, suggesting that there are other pathways that may also contribute to this cytokine production. In fact, EFL2 did inhibit NF-κB signaling pathway activation (Figure 6). More importantly, we also showed that NF-κB signaling activation is important to TLR7-mediated IL-1β production. Taken together, our data suggest that EFL2 exerts anti-inflammatory effect by suppressing TLR7-induced IRAK4-IKKβ-IRF5 and NF-κB signaling pathways activation.

Targeting TLRs ligands or the relevant signaling pathways is a promising strategy for rheumatoid arthritis therapy. Here, we demonstrate that EFL2 exerts the robust anti-arthritic effect by inhibiting myeloid cells infiltration and IL-1β and IL-6 production whereby TLR7-mediated IRAK4-IKKβ signaling activation were significantly blocked. Moreover, we identified EFL2 as an effective IRF5 inhibitor to block the inflammatory responses during arthritis development. This novel information indicates the new opportunity for further investigation of EFL2 as a potential candidate in TLR7 or IRF5-dependent inflammatory diseases.

## Data Availability

The original contributions presented in the study are included in the article/Supplementary Material, further inquiries can be directed to the corresponding author.

## Supplementary Material

Supplementary Figure S1 Euphorbia factor L2 suppresses inflammatory cells expansion in the peripheral blood. Representative flow cytometry plots and quantification of the percentages of neutrophils (Ly6G+) **(A,B)**, macrophages (CD11b+F4/80+) **(C,D)**, and inflammatory monocytes (CD11b+Ly6C+Ly6G−) **(E,F)** in the blood from the control, STA, STA mice treated with DEX or EFL2 (15 or 40 mg/kg) and mice solely treated with EFL2 (40 mg/kg) (n = 3−7 for each group). Data represent the mean values ± SEM of three independent experiments. ***p*<0.05, ***p*<0.01 were considered statistically significant.

Supplementary Figure S2 IRF5 expression in RAW 264.7 cells after transfected with IRF5 plasmid. **(A)** Western blot bands **(B)** Quantification of IRF5 expression in R837-stimulated RAW264.7 cells before and after IRF5 overexpression. Data represent the mean values ± SEM of three independent experiments. *p<0.05, **p<0.01, ***p<0.001 were considered as significant.
