# Supplementary figures and images for: Euphorbia Factor L2 ameliorates the Progression of K/BxN Serum-Induced Arthritis by Blocking TLR7 Mediated IRAK4/IKKβ/IRF5 and NF-kB Signaling Pathways
Source: Front Pharmacol. 2021 Dec 3;12:773592. doi: 10.3389/fphar.2021.773592 (PMC8691750; doi:10.3389/fphar.2021.773592)

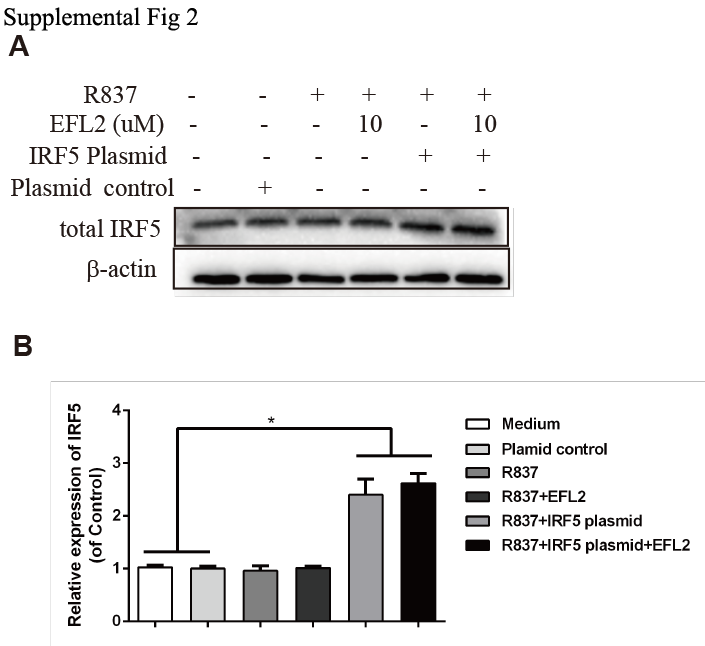

Supplement: Supplementary file 1 [file Image2.TIF]

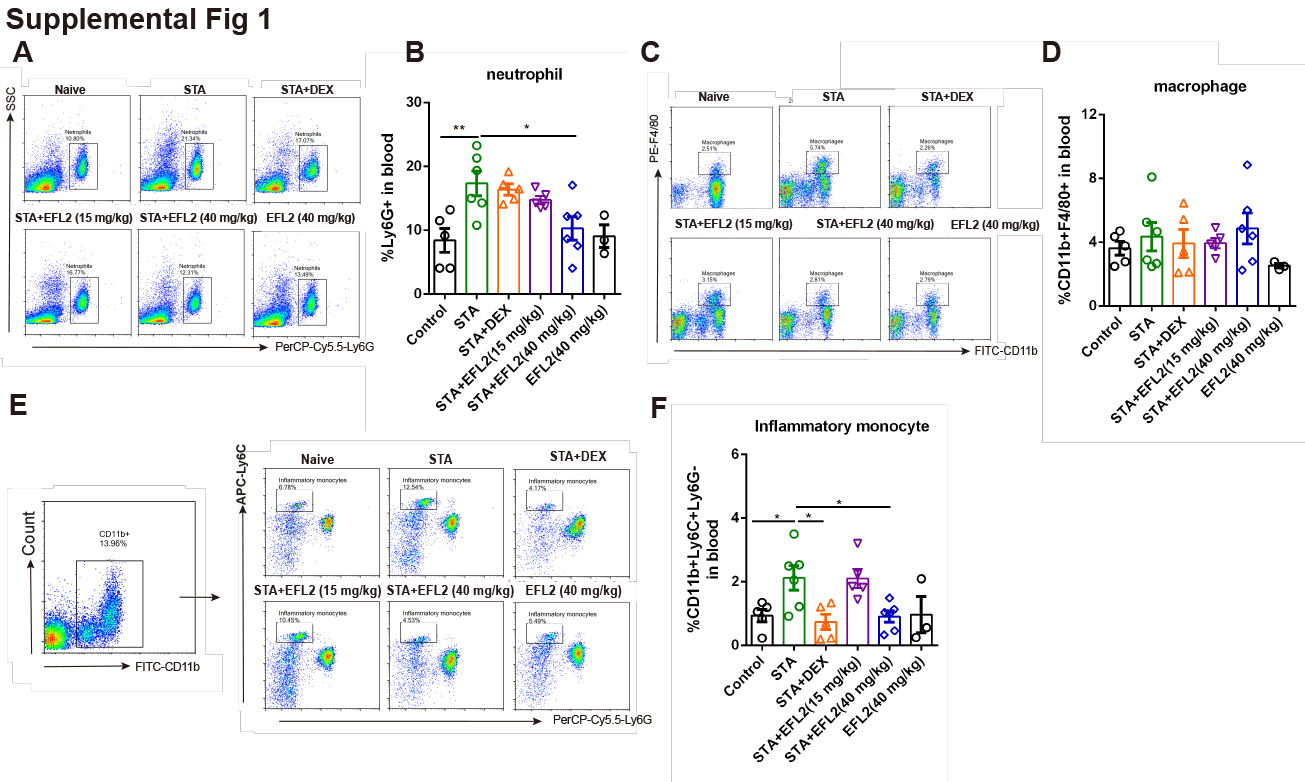

Supplement: Supplementary file 2 [file Image1.TIF]
